# Neonatal acute liver failure cases with echovirus 11 infections, Japan, August to November 2024

**DOI:** 10.2807/1560-7917.ES.2025.30.1.2400822

**Published:** 2025-01-09

**Authors:** Tatsuki Ikuse, Toshihiro Matsui, Kensuke Shoji, Naoko Kono, Masaki Yamada, Chikara Ogimi, Chika Takahashi, Takanori Funaki, Kentaro Ide, Shotaro Matsumoto, Reiko Ito, Rinshu Shimabukuro, Yoshihiro Gocho, Itaru Hayakawa, Takashi Ishikawa, Seisuke Sakamoto, Mureo Kasahara, Takashi Igarashi

**Affiliations:** 1 Division of Infectious Diseases, Department of Medical Subspecialties, National Center for Child Health and Development, Tokyo, Japan; 2 Infectious Disease Control Division, Setagaya Public Health Center, Tokyo, Japan; 3 Center for Research Planning and Coordination, National Institute of Infectious Diseases, Tokyo, Japan; 4 Critical Care Medicine, National Center for Child Health and Development, Tokyo, Japan; 5 Department of General Paediatrics and Interdisciplinary Medicine, National Center for Child Health and Development, Tokyo, Japan; 6 Children's Cancer Center, National Center for Child Health and Development, Tokyo, Japan; 7 Division of Neurology, National Center for Child Health and Development, Tokyo, Japan; 8 Division of Immunology, National Center for Child Health and Development, Tokyo, Japan; 9 Organ Transplantation Center, National Center for Child Health and Development, Tokyo, Japan; 10 President’s office, National Center for Child Health and Development, Tokyo, Japan

**Keywords:** enterovirus, echovirus 11, acute liver failure, hepatitis, neonate

## Abstract

In 2022–23, several European countries reported paediatric acute liver failure (ALF) with enterovirus infection. In August–November 2024, three neonatal cases of ALF with echovirus 11 (E11) were reported in Tokyo, Japan. All neonates developed irreversible multiple-organ failure and died. The E11 strain belonged to the new lineage 1, which was the same as strains isolated from neonatal ALF cases in Europe in 2022–23.

As the COVID-19 pandemic waned, the Japanese government lifted the requirement for strict public health measures by May 2023 [1]. Subsequently, paediatric viral infections have increased, including invasive infections caused by echovirus 11 (E11) and other enteroviruses (EVs) in neonates. Since 2022, a new E11 lineage (new lineage 1) has been reported as a cause of neonatal severe acute liver failure (ALF) in Europe [2-4].

Here, we describe three neonatal cases of invasive E11 infections in Japan between August 2024 and November 2024 and analyse E11 lineage to assess the relationship between local E11 strains and those reported in Europe.

## Identification of paediatric cases of acute liver failure

The National Center for Child Health and Development (NCCHD), one of the largest tertiary children’s hospitals in Tokyo, Japan, provides intensive care for critically ill patients, including severe cases referred from across the country. Additionally, NCCHD is a major centre for liver transplantation, and therefore receives referrals for paediatric ALF cases potentially requiring transplantation.

Between 1 August 2024 and 30 November 2024, we managed three neonates presenting with ALF and multi-organ failure (MOF). We performed real-time multiplex PCR using FTD Neuro 9 (Fast Track Diagnostics Ltd., Esch-sur-Alzette, Luxembourg), which targets EV, parechovirus, herpes simplex 1 and 2, cytomegalovirus, Epstein-Barr virus, adenovirus, varicella-zoster virus, human herpes viruses 6 and 7, and parvovirus B19, on serum samples as a routine diagnostic procedure for ALF cases, and genotyping was conducted for EV-positive samples using the CODEHOP approach [5]. Sequencing and genotyping were performed with BLAST (https://blast.ncbi.nlm.nih.gov/Blast.cgi). Lymphocyte subset analysis was performed by flow cytometry to screen for inborn errors of immunity and assess the presence of activated natural killer (NK)/T-cells.

## Acute liver failure case characterisation

Symptom onset for the three neonates with ALF and MOF occurred between 7 and 8 days of age. Initial symptoms included jaundice, poor feeding and lethargy, with disease progressing rapidly (1–2 days from symptom onset) and necessitating transfer to the paediatric intensive care unit after admission. Two of the three neonates met the diagnosis criteria for haemophagocytic lymphohistiocytosis (HLH) [6], a life-threatening condition characterised by an overactivation of the immune system, and treatment for HLH was initiated. In all three cases, lymphocyte subset analysis revealed no abnormalities other than mild activation of T-cells and slight elevation of soluble interleukin-2 receptor. These findings suggested that an underlying disease causing HLH was unlikely. All neonates developed irreversible MOF and died. Clinical and genetic characteristics of the three cases are presented in the Table and detailed clinical courses of each case are summarised in the Supplement. 

**Table:** Neonatal cases of acute liver failure with enteroviral infection, Japan, August–November 2024 (n = 3)

Characteristics	Case 1	Case 2	Case 3
Clinical background			
Month of onset in 2024	August	August	November
Age at onset (days)	7	8	8
Gestational age (weeks + days)	38 + 3	37 + 0	37 + 4
Birth weight (g)	2,674	2,454	2,300
Sex	Male	Male	Male
Delivery	Normal vaginal delivery	Caesarean section	Caesarean section
Sick contacts	Father (fever)	None	None
Diagnosis	ALF, HLH, Torsades de pointes, renal failure	ALF, HLH, renal failure	ALF, renal failure
Known underlying diseases	None	None	None
Outcome	Died	Died	Died
Laboratory results			
Serum PCR^a^	EV	EV	EV
EV genotype (GenBank accession number)	Echovirus 11 (LC849101)	Echovirus 11 (LC855180)	Echovirus 11 (LC855181)
FilmArray respiratory panel^b^	Not performed	HRV/EV	HRV/EV

ALF: acute liver failure; EV: enterovirus; HLH: haemophagocytic lymphohistiocytosis; HRV: human rhinovirus.

^a^ The serum samples were analysed by real-time multiplex PCR using FTD Neuro 9 including enterovirus (EV) detection.

^b^ The FilmArray™ Respiratory Panel 2.1 (RP2.1) (BioMérieux, Saint Louis, United States) is a rapid diagnostic tool that identifies various respiratory pathogens using PCR technology. It works with the BIOFIRE FILMARRAY system to detect both viral and bacterial pathogens in nasopharyngeal swabs from individuals suspected of having respiratory infections. In our institution, the FilmArray™ RP2.1 was not routinely employed for pathogen analysis in patients with ALF. Instead, its use was determined on a case-by-case basis by the attending clinician in the emergency department.

Presence of EV was confirmed in serum samples from all three cases via multiplex real-time PCR, whereas other viruses were negative. Molecular analysis identified E11. 

## Molecular and phylogenetic analyses

**Figure 1:**
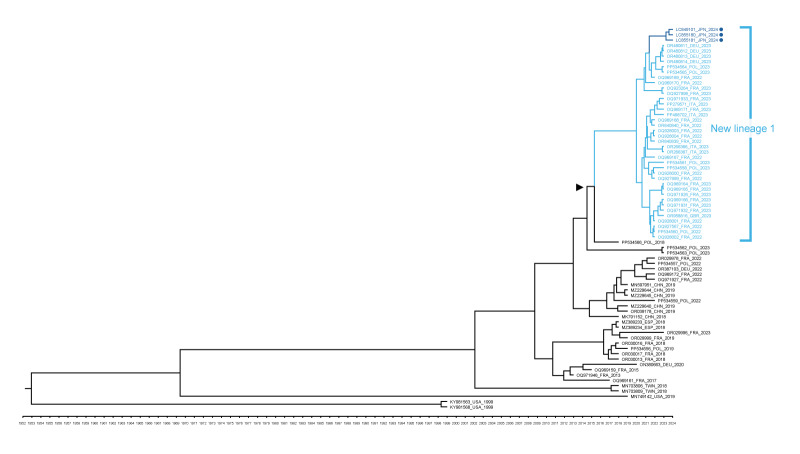
Phylogenetic tree of echovirus 11 sequences detected in Japan (n = 3), 2024 and other countries (n = 69), 1999–2024

Phylogenetic analysis using the Bayesian Markov chain Monte Carlo method was performed on partial VP1 region sequences from E11 strains previously reported from Europe, the United States and China (n = 69) and from the three strains in this report (accession numbers: LC849101, LC855180, and LC855181 in the NCBI GenBank database) [2,3,7,8]. The E11 strains from the ALF cases belong to the new lineage 1 within the D5 genotype, which includes strains detected in severe neonatal cases in Europe between 2022 and 2023 (Figure 1) [2,3,7,8]. The mean time to the most recent common ancestor (TMRCA) of the new lineage 1 was estimated to be 2016. Detailed methods are described in the Supplement.

Phylogenetic analysis using the Bayesian Markov chain Monte Carlo method was performed using partial VP1 regions of echovirus 11 (E11) detected in Japan (n = 3) and other countries (Europe, US and China) (n = 69). The BEAST software package BEAST version 2.6.7 was used to obtain estimated evolutionary rates. The effective sample size for each estimate was confirmed to be greater than 200 (minimum, 724) using Tracer version 1.7.2. A maximum clade credibility tree was constructed using TreeAnnotator version 2.6.6 after a 10% burn-in. The tree was constructed using FigTree version 1.4.4. Each strain was described as ‘accession number_ country detected _ year detected’. Blue lines indicate the E11 new lineage 1, and the three sequences detected in Japan are listed in dark blue in the tree. The mean substitution rate was 4.44 × 10^−3^ (95% highest probability density, 2.87 × 10^−3^–6.03 × 10^−3^) per site per year. The arrow indicates the branching point of the new lineage 1. The mean time to the most recent common ancestor (TMRCA) of the diverse new lineage 1 was estimated to be 2016.

## Enterovirus PCR results for acute liver failure cases hospitalised in 2022–2024

**Figure 2:**
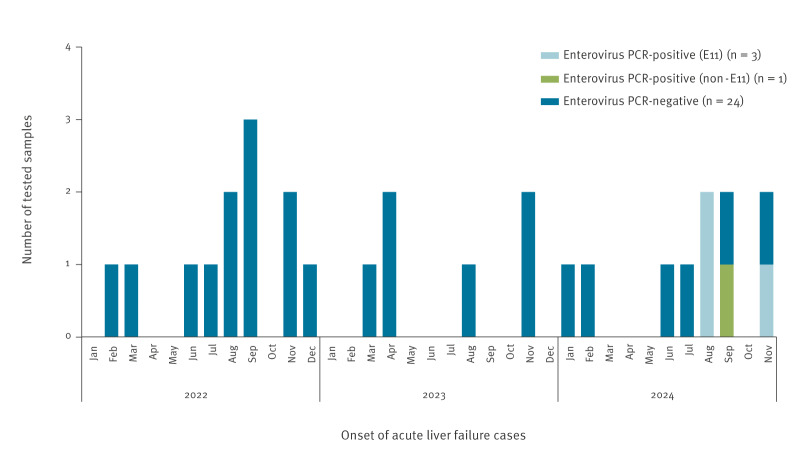
Results of enterovirus real-time PCR for acute liver failure cases, Japan, January 2022–November 2024 (n = 28)

A diagnostic work-up for inpatient ALF cases at the NCCHD was conducted longitudinally. Enterovirus real-time PCR was performed on serum samples collected from 12 patients with ALF in 2022, 6 in 2023 and 10 in 2024. The age range was 0–12 years old. Among them, EV was detected only in four of the 10 cases in 2024. E11 was identified in two cases in August and one case in November 2024. Coxsackievirus B4 was identified in one case in September 2024 (Figure 2).

Serum samples of acute liver failure cases were analysed with a multiplex real-time PCR assay that included enterovirus (EV) detection.

## National surveillance of echovirus 11 in Japan in 2022–2024

**Figure 3:**
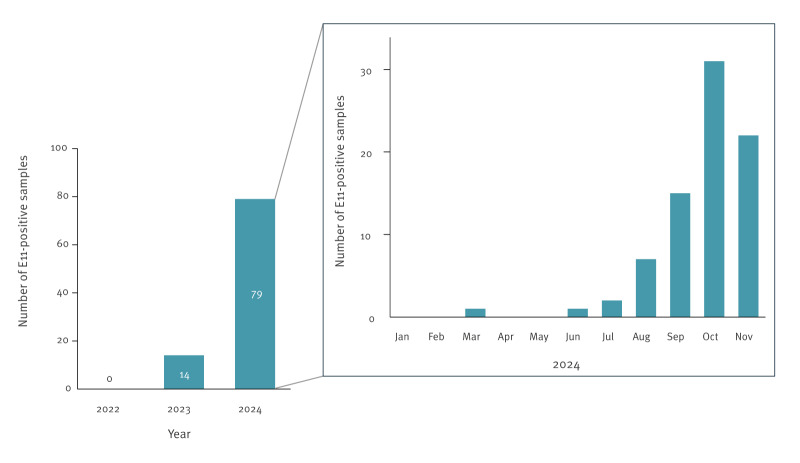
National surveillance of echovirus 11 in Japan, January 2022–November 2024 (n = 93)

In Japan, the National Epidemiological Surveillance of Infectious Diseases (NESID) was initiated in April 1999 under the Infectious Disease Control Law. Under this law, sentinel laboratory-based surveillance including that for enteroviruses was also conducted using specimens from medical facilities designated by each prefecture. The NESID in Japan reported a remarkable increase in E11 cases in 2024 (n = 79), mostly between August and November (Figure 3).

In Japan, the National Epidemiological Surveillance of Infectious Diseases (NESID) is conducted to determine the comprehensive epidemiological trends of pathogens, including echovirus 11 (E11) (https://www.niid.go.jp/niid/ja/iasr/511-surveillance/iasr/tables/1493-iasrtv.html. Accessed on 26 December 2024). The number of detected specimens of E11 increased in 2024, particularly between August and November.

## Discussion

We identified three neonates in Japan with fatal ALF and infection with the E11 new lineage 1, aligning with its reported circulation in Europe since 2022 and association with severe neonatal ALF [2-4].

Echovirus 11 is responsible for notable global outbreaks, causing a wide range of clinical symptoms, particularly in neonates and infants [9]. In neonates, E11 complications include meningitis, myocarditis, and fulminant hepatitis [9]. Herein, the three neonates with ALF and MOF that had fatal outcomes were male, consistent with a previous report from France [2]. Although B-cell deficiency was reported as a risk of severe EV infection [10], flow cytometry-based screening for inborn errors of immunity showed no remarkable abnormalities. However, a review by Zhang et al. implied that young age and male sex are risk factors for fatal outcomes among severe neonatal enteroviral infection cases [11]. Moreover, low levels of transplacental maternal antibodies against the new lineage 1 might have made these cases more susceptible to rapidly progressive E11 infections than to other EVs [12].

Between 2022 and 2023, an increase in severe cases caused by a new highly pathogenic E11 variant (new lineage 1) was reported in Europe, prompting a World Health Organization alert [2-4,7,8]. While our phylogenetic analysis using VP1 region estimated TMRCA of the new lineage 1 to be from 2016, another phylogenetic analysis based on a different genomic region estimated TMRCA to be from 2018 [13]. These findings suggest that the new lineage 1 emerged before the COVID-19 pandemic, although its circulation has been ongoing since the pandemic onset.

In 2023, the E11 strain was also reported to be circulating in China [9]. Since the circulation occurred after the emergence of the new lineage 1 in Europe, the spread of the same lineage in China was suspected. However, this E11 lineage detected in China, called ‘D5-CHN2’ and different from the new lineage 1, was identified through phylogenetic analysis and was estimated to have spread from China to Europe by 2023 [9]. The spread of D5-CHN2 from China to Japan was possible, owing to the geographic proximity. However, the E11 strains of the three cases in our hospital belonged to new lineage 1, clustering with sequences from Germany and Poland in 2023. These findings suggest a spread of new lineage 1 from France and Italy to Germany and Poland, and then Japan.

During the COVID-19 pandemic, the incidence of most respiratory viral infections declined because of the non-pharmaceutical interventions [14]. However, as infection control measures were relaxed, they resurged [15]. This trend was reflected by shifts in the epidemiology of various EV infections in response to changes in infection control strategies [15]. In addition, in 2024, with a record-high number of inbound tourists, there is a high possibility that viruses currently spreading overseas may be brought into Japan by tourists [16]. 

Based on the increase of E11, the Ministry of Health, Labour and Welfare (on 3 December2024) and the Japan Paediatric Society (on 1 December 2024) issued an alert regarding a potential epidemic of E11-related severe infections in newborns [17,18]. Following the alert, the National Institute of Infectious Diseases in Japan summarised data on E11 cases between January 2018 and November 2024 [19]. In 2024, 44 cases were included (neonates accounted for 13.6%, while infants aged 1–11 months comprised 31.8%). The most common diagnoses were aseptic meningitis, gastroenteritis and upper respiratory infections. E11 circulating in Japan in 2024 caused diverse clinical presentations, including severe ALF in children. In a report from Poland, most patients with E11 had mild presentations, while only one was a neonate with severe hepatitis [20]. Similarly, in Spain, most neonates infected with E11 new lineage 1 presented with meningoencephalitis and the majority did not require ICU management. Cases with known outcomes (n = 7) survived [7].

## Conclusion

Our report underscores the emergence of the highly pathogenic E11 new lineage 1 in Japan, which has been associated with fatal neonatal cases of ALF and MOF. These findings are consistent with similar reports from some countries in Europe, highlighting the possible severe clinical impact of this lineage. Continuous surveillance and molecular analysis of neonatal EVs are necessary to track their global dissemination and understand their clinical manifestations and epidemiology.

## Supplementary Data

263000 Supplement
