# Supplementary material for: Neonatal acute liver failure cases with echovirus 11 infections, Japan, August to November 2024
Source: Euro Surveill. 2025 Jan 9;30(1):2400822. doi: 10.2807/1560-7917.ES.2025.30.1.2400822 (PMC11719806; doi:10.2807/1560-7917.ES.2025.30.1.2400822)
Supplement: Supplement [file 24-00822_SHOJI_Supplement.pdf]

Supplement

This supplementary material is hosted by *Eurosurveillance* as supporting information alongside the article, titled 'Neonatal acute liver failure cases with echovirus 11 infections, Japan, August to November 2024', on behalf of the authors, who remain responsible for the accuracy and appropriateness of the content. The same standards for ethics, copyright, attributions, and permissions as for the article apply. Supplements are not edited by *Eurosurveillance* and the journal is not responsible for the maintenance of any links or email addresses provided therein.

Contents:

1) Detailed clinical courses of three acute liver failure cases
2) Method of molecular and phylogenetic analyses

Classified as ECDC NORMAL

1) Detailed clinical courses of three acute liver failure (ALF) cases

1-1) Case 1

At 7 days of age, the neonate presented with jaundice, somnolence, and poor feeding and was brought to the referring hospital because of persistence of the symptoms on day 9 of life. The neonate was transferred to the paediatric intensive care unit (PICU) of the National Centre for Child Health and Development (NCCHD) because of ALF, renal failure, and haemophagocytic lymphohistiocytosis (HLH). On day 15 of life, the patient developed torsades de pointes. On day 27 of life, the patient had generalised seizures; however, imaging of the brain and a lumbar puncture (LP) could not be performed because of an altered general status and abnormal coagulation. Although intensive care for ALF and HLF was continued, multi-organ failure persisted. On day 32 of life, sudden circulatory collapse occurred, accompanied by intestinal pneumatosis and bacteraemia with *Enterococcus faecalis* and *Klebsiella oxytoca*. The neonate died on the same day due to refractory septic shock.
Regarding viral analysis, real-time polymerase chain reaction (PCR) for enterovirus (EV), parechovirus (PeV), and others using FTD Neuro 9™ (Fast Track Diagnostics Ltd.) was performed using serum collected on day 9 of life. Only the test for EV was positive. Echovirus (E11) was identified using BLAST analysis.

1-2) Case 2

On day 8 of life, the neonate was unwell, fed poorly, and was brought to a hospital. Nasopharyngeal swabs were positive for human rhinovirus/EV using FilmArray™ respiratory panel version 2.1 (Biomerieux, Saint-Louis, US). The FilmArray™ meningitis/encephalitis panel test result was negative. The neonate was diagnosed with ALF, renal failure, and HLH, and showed a poor response to intensive care. Due to severe liver failure, the patient was transferred to the PICU at the NCCHD for liver transplantation. However, controlling HLH is difficult, and there is no indication for liver transplantation due to multi-organ failure. The patient died on day 52 of life. Real-time PCR for EV, PeVs, and others using FTD Neuro 9™ was performed on the serum collected on the first day of admission (8 days old) in the referring hospital. Only EV was positive, and E11 was identified. Genetic testing for familial HLH showed no pathologic variants.

1-3) Case 3

On day 8 of life, the patient was vomiting and was admitted to the referring hospital, presenting with weight loss, lethargy, thrombocytopenia, anaemia, elevated AST and ALT levels, hyperferritinaemia, and oliguria.
The patient was transferred to the PICU at the NCCHD on day 9, where continuous haemodiafiltration, plasma exchange, and intravenous immunoglobulin therapy were initiated. Despite interventions, renal failure progressed, requiring renal replacement therapy. An indication of liver transplantation was considered due to EV11-driven irreversible end-stage liver disease; however, the patient died due to septic shock caused by bacteraemia of *Staphylococcus aureus* and *Staphylococcus epidermidis* without any focus on day 25. On day 10, viral analysis using Real-time PCR with FTD Neuro 9™ on serum was EV positive, and BLAST analysis confirmed E11.

2) Method of molecular and phylogenetic analyses

For phylogenetic analysis using the Bayesian Markov chain Monte Carlo (MCMC) method, we used partial VP1 region sequences for the E11 strains (n = 69) used in previously reported phylogenetic analyses. The partial VP1 regions of the current EV11 strains (three cases of ALF) were deposited in the NCBI GenBank database (accession numbers LC849101, LC855180, and LC855181). Using the Molecular Evolutionary Genetics Analysis software version 6 (MEGA6), after multiple alignments with the ClustalW program, we selected the Hasegawa-Kishino-Yano model with gamma distribution as the best substitution model. The BEAST software package BEAST version 2.6.7 was used to obtain estimated evolutionary rates. A lognormal relaxed molecular clock and constant population size coalescent model with a chain length of 30 million were used for the analysis. The effective sample size for each estimate was confirmed to be greater than 200 (minimum, 724) using Tracer version 1.7.2. A maximum clade credibility tree was constructed using TreeAnnotator version 2.6.6 after a 10% burn-in. The tree was constructed using FigTree version 1.4.4. The year of each virus strain corresponded to the date of sample collection. The mean time to the most recent common ancestor (TMRCA) of the diverse new lineage 1 (►) was estimated (Figure 1).

Classified as ECDC NORMAL
